# Pyridoxine biosynthesis protein MoPdx1 affects the development and pathogenicity of *Magnaporthe oryzae*

**DOI:** 10.3389/fcimb.2023.1099967

**Published:** 2023-02-07

**Authors:** Lina Yang, Xiaohong Liu, Jie Wang, Lianwei Li, Wanzhen Feng, Zhaolin Ji

**Affiliations:** ^1^ College of Plant Protection, Yangzhou University, Yangzhou, Jiangsu, China; ^2^ The Key Laboratory of Biotechnology for Medicinal Plants of Jiangsu Province, School of Life Science, Jiangsu Normal University, Xuzhou, Jiangsu, China; ^3^ Hainan Yazhou Bay Seed Laboratory, Sanya Nanfan Research Institute of Hainan University, Sanya, China

**Keywords:** *Magnaporthe oryzae*, MoPdx1, vitamin B6, development, pathogenicity

## Abstract

B vitamins are essential micro-organic compounds for the development of humans and animals. Vitamin B6 comprises a group of components including pyridoxine, pyridoxal, and pyridoxamine. In addition, vitamin B6 acts as the coenzymes in amino acid biosynthesis, decarboxylation, racemic reactions, and other biological processes. In this study, we found that the expressions of a gene encoding pyridoxine biosynthesis protein (*PDX1*) were significantly upregulated in the early infectious stages in *M. oryzae*. Furthermore, deletion of *MoPDX1* slowed vegetative growth on different media, especially on MM media, and the growth defect was rescued when MoPdx1-protein was expressed in mutants strains and when commercial VB6 (pyridoxine) was added exogenously. However, VB6 content in different strains cultured in CM media has no significant difference, suggested that MoPdx1 was involved in *de novo* VB6 biosynthesis not in uptake process, and VB6 regulates the vegetative growth of *M. oryzae*. The Δ*Mopdx1* mutants presented abnormal appressorium turgor, slowed invasive growth and reduced virulence on rice seedlings and sheath cells. MoPdx1 was located in the cytoplasm and present in spore and germ tubes at 14 hours post inoculation (hpi) and then transferred into the appressorium at 24 hpi. Addition of VB6 in the conidial suspentions could rescue the defects of appressorium turgor pressure at 14 hpi or 24 hpi, invasive growth and pathogenicity of the *MoPDX1* deletion mutants. Indicated that MoPdx1 affected the appressorium turgor pressure, invasive growth and virulence mainly depended on *de novo* VB6, and VB6 was biosynthesized in conidia, then transported into the appressorium, which play important roles in substances transportation from conidia to appressorium thus to regulate the appressorium turgor pressure. However, deletion of *MoPDX1* did not affect the ability that scavenge ROS produced by rice cells, and the mutant strains were unable to activate host defense responses. In addition, co-immunoprecipitation (Co-IP) assays investigating potential MoPdx1-interacting proteins suggested that MoPdx1 might take part in multiple pathways, especially in the ribosome and in biosynthesis of some substances. These results indicate that vitamins are involved in the development and pathogenicity of *M. oryzae*.

## Introduction

As one of the world’s leading food crops, rice (*Oryza sativa*) has long been affected by rice diseases, that can result in large-scale production reduction. Rice blast is one of the most significant and devastating diseases in rice production, causing losses of 10% to 30% every year (Skamnioti and Gurr, 2009; Sakulkoo et al., 2018). Due to the rapid variation of field strains of the disease, resistant varieties of rice lose their resistance quickly after a few years of planting. Meanwhile, large-scale pesticide use readily causes environmental pollution and residues. Therefore, analyzing the pathogenic mechanism of the rice blast fungus *Magnaporthe oryzae* could provide candidate targets for developing new fungicides and offer new strategies for preventing and controlling rice blast.

The infection cycle of *M. oryzae* was composed of four diverse stages. Initially, conidia sense hydrophobic surface signals and adhere to the host surface by secreting spore tip mucilage (Wilson and Talbot, 2009). The second stage is spore germination and development of the germ tube, then forms the appressorium. The third stage is formation of penetration peg. With the maturation of the appressorium, enormous turgor pressure, approximately 8~10 MPa, develops, providing a massive mechanical force to penetrate the host cuticle and enter the host cells (Howard et al., 1991; Foster et al., 2017). Finally, infectious hyphae disrupt the nutrients and water of the host and secret effectors to suppress plant immunity (Giraldo et al., 2013). Further, they spread into the neighboring host cells, and eventually, the host cells die (Jia et al., 2016; Gupta et al., 2021).

B vitamins are essential micro-organic compounds for the development of humans and animals, and consist of 8 kinds of water-soluble complex vitamins, including vitamins B1, B2, B3, B5, B6, B7, B9, and B12 (Kennedy, 2016; Calderón-Ospina and Nava-Mesa, 2020). All of the B vitamins act synergistically to regulate metabolism, enhance functions of the nervous system and immune systems, and promote cell growth and division (Kennedy, 2016; Wendołowicz et al., 2018). Among them, VB6 comprises a group of compounds including pyridoxine, pyridoxal, and pyridoxamine. Pyridoxamine is the aminated product of pyridoxine, and pyridoxal is the formyl derivative of pyridoxine. Pyridoxal, pyridoxine, and pyridoxamine have equal activities in animals. In general, pyridoxal, pyridoxine, and pyridoxamine are converted into the enzymatically active forming pyridoxal-5-phosphate (PLP), pyridoxine-5-phosphate (PNP), and pyridoxamine-5-phosphate (PMP), respectively, which carry out their vitamin activities (Rosenberg, 2012). More than 160 enzymes with different catalytic activities have been identified that require VB6 (particularly PLP) as coenzymes to function (approximately 4% of all catalytic activity) (Percudani and Peracchi, 2009); PLP-dependent enzymes take part in amino acid biosynthesis, decarboxylation, and racemic reactions (Eliot and Kirsch, 2004). PLP regulates the activity of the transcription factor PdxR, and feedback regulates the biosynthesis of VB6 in *Streptococcus pneumonia* (El Qaidi et al., 2013). Together, these studies suggest that VB6 plays multiple functions in different cellular processes.

VB6 can be synthesized by plants, fungi, bacteria, archaea, and protists. However, animals and some obligate pathogens require it only from foods (Eliot and Kirsch, 2004; Phillips, 2015). In *Arabidopsis*, deletion of pyridoxine biosynthesis protein (*de novo* vitamin B6 biosynthesis protein, *PDX1*) leads to abnormal development of the roots and seedlings, and the mutants were more sensitive to osmotic stress and oxidative stress, suggesting that VB6 might be an antioxidant (Chen and Xiong, 2005). In tomatoes, SlPdx1.2 and SlPdx1.3 regulate the antioxidant capacity of cells, thus cofferring resistantance to *Botrytis cinerea* infection (Zhang et al., 2014). In rice, pyridoxal phosphate synthase OsPdx1 positively regulated rice immunity to *Xanthomonas oryzae pv. oryzicola* (*Xoc*), inducing stomatal closure by promoting the biosynthesis of abscisic acid (ABA), which is targeted and degraded by the effector protein AvrRxo1 of *Xoc* (Liu et al., 2022). In fungi, the functions of Pdx1 have only been reported for *Cercospora nicotianae*, one of plant pathogens. Cercosporin, the main pathogenic factor generated by *C. nicotianae*, could absorb light and interact with oxygen to produce superoxide ions ( ${\text{O}}_{2}^{-}$ ), hydrogen peroxide (H_2_O_2_), highly destructive singlet oxygen (^1^O_2_), and other substances to promote host infection. In addition to function in the biosynthesis of VB6, Pdx1 of *C. nicotianae* is also a ^1^O_2_ resistance proteins involved in the resistance process of cercosporin and other photosensitizers, and in protecting itself from reactive oxygen species (ROS) (Ehrenshaft et al., 1999). Furthermore, Pdx1 was found to be located in vesicles of *C. nicotianae* (Chung et al., 2002). However, the molecular mechanisms of Pdx1 in its antioxidant capacity and vesicle localization are still unknown. In addition, there are no known functions of Pdx1 in the virulence of plant pathogens.

In this work, the expression of *PDX1* was significantly upregulated at the early infectious stages compared with the hyphal stage in *M. oryzae*. Furthermore, the *MoPDX1* was deleted, and it was found that the vegetative growth of mutants was significantly slower in MM media (uncomplete media) than in CM and SDC media, and exogenous VB6 (pyridoxine) could rescue the growth defects. We found that MoPdx1 was involved in the pathogenicity of *M. oryzae* mediating *de novo* biosynthesis of VB6. Besides that, a Co-IP assay was carried out to identify its potential interacting protein and thereby investigate the functions of Pdx1 in *M. oryzae*.

## Materials and methods

### Strains and culture conditions

The strain Guy11 (isolated from Guyana, which has relatively higher virulent) was used as a wild-type in this work. The Guy11, mutants and complementary strains were cultured at 28°C in CM, MM, and SDC media for 7 days, respectively. Fungal mycelia were harvested after growth in liquid CM media with shaking for 48 h at 28°C and were used for DNA and RNA extraction. Fungal protoplasts were prepared and transformed as described previously (Sweigard et al., 1992). Transformants were selected on TB3 media with hygromycin B or zeocin (Yang et al., 2019). For conidiation statistics, mycelial blocks were cultured on SDC media for 7 days in the dark and then moved to constant illumination under fluorescent light for 3 days (Zhang et al., 2009).

### Target gene deletion and complementary strains obtained in *M. oryzae*

The approximately 1.0 kb up- and downstream- sequences of *MoPDX1* (MGG_05980) were separately amplified from *M. oryzae* genomic DNA with the primers shown in **Table S1**. They were ligated to the pCX62 vectors in two steps to generate the *MoPDX1* deletion vectors, which were confirmed by sequencing. Then, the deletion vectors were transformed into the Guy11 protoplasts, which were selected on TB3 media supplemented with hygromycin B. Candidate transformants were verified with inner and outer primers (in **Table S1**) of *MoPDX1* through PCR and further verified by Southern blot analysis (**Figure S1**). For complementary strains, fragments including the full-length coding region without the termination code and approximately 1.5 kb native promoters were cloned into the pYF11 vectors, which express green fluorescence protein (GFP), and sequenced. Then, the complementary vectors were transformed into the *MoPDX1* deletion mutants and selected by zeocin-resistance. Candidate strains were verified by GFP screening and Western blot assays.

### Vitamin supplementation assay

To test the function of vitamins in the development of *M. oryzae*, hyphal blocks of Guy11, two Δ*Mopdx1* mutants, and complementary strains were cultured on MM media supplemented with 10^-4^ mg/mL of VB1 (Vitamin B1, Cat#V8020, Solarbio), VB2 (Riboflavin, Cat#V8060, Solarbio), VB3 (Nicotinic acid, Cat#N8060, Solarbio), VB6 (Pyridoxine HCl, Cat#V8030, Solarbio), VH (D-biotin, Lot F801BA0013, Sangon Biotech, Shanghai, China), or PABA(4-aminobenzoic acid, Lot F906BA0033, Sangon Biotech, Shanghai, China) alone or lacking only VB1, VB3, and VB6, at 28°C for 7 days in the dark. The hyphal blocks of the indicated strains cultured on MM media were used as controls. For the invasive growth complementation assay, 10^-4^ mg/mL of VB6 were added in the conidial suspensions of the *MoPDX1* deletion mutants, then injected into the 21-d rice sheath cells, results were observed at 24 and 48 hours post inoculation (hpi), respectively. For the virulence complementation assay, 25 μL of conidial suspensions (5×10^4^ spores/mL) of mutants supplemented with 10^-4^ mg/mL VB6 were inoculated on the surface of 7-day barely leaves. For the appressorium turgor complementation assay, 10^-4^ mg/mL of VB6 were added in the conidial suspensions of indicated strains, 25 μL of them were cultured on the hydrophobic slides (Fisherbrand, 12540A, Germany), different concentrations of 1-4 M glycerol were used to treat the appressoria for 3 min to detect the their incipient cytorrhysis. At least 100 appressoria were counted at 14 and 24 hpi for each experiments, respectively.

### Intracellular VB6 measurements

The indicated strains were reactivated from PDA media and cultured on CM media at 28°C for 5 d in the dark. Then, the samples were shaken in liquid CM media for 48 h, and the mycelium pellets were collected and quickly ground into powders in liquid nitrogen. The material extraction method was described in the previous study (Liu et al., 2016a). Intracellular VB6 in different samples were detected by HPLC-MS (Agilent 6460, USA) analysis. The steps were as follows: at 0~5 min, the C18 column (2.1× 50 mm, 2.7 μm; Agilent, USA) was rinsed with 100% water (pH 3.0-4.0) containing 5 mM ammonium acetate; at 6~7 min, the C18 column was rinsed with 100% acetonitrile; and at 8~15 min, the C18 column was rinsed again with 100% water (pH 3.0-4.0) containing 5 mM ammonium acetate. The flow rate was 0.2 mL per minute, and the temperature of the C18 column was 35°C. The peak flow of the VB6 standard was at approximately1.25 min. The intracellular VB6 content in different samples was calculated according to a previously described method (Liu et al., 2016a).

### Appressorial formation, turgor pressure, and rice infection assays

For appressorial formation, spores of the indicated strains were collected with a double filter cloth, and 20 μL of a 5×10^4^ spores/mL suspensions were dripped onto hydrophobic slides. At least 100 appressoria were counted at 24 hpi. For turgor pressure, 1-4 M glycerol was used to treat the appressoria for 3 min at 14 and 24 hpi, and at least 100 appressoria were counted to observe their collapse rate. For rice spray assays, 5×10^4^ spores/mL conidia suspensions of the indicated strains were sprayed onto the surface of 11-d rice CO-39 (a susceptible variety to rice blast) seedlings. After incubation in a moist chamber at 28°C for 24 h in the dark, the samples were transferred into another moist chamber at 28°C for 6 days under continuous12 h/12 h light and dark conditions. For rice leaf sheath injection assays, 15×10^4^ spores/mL conidial suspensions were injected into 21-d rice leaf sheaths, and the conditions of disease occurrence were consistent with those of the rice spray assay. The experiments were repeated three times with the same results.

### Quantitative reverse transcription PCR (qRT–PCR) analysis

For expression analysis assays of *MoPDX1* at different stages of *M. oryzae*, hyphal blocks of Guy11 strains were cultured in liquid CM media for 48 h at 28°C, conidia of Guy11 were collected in SDC media, 5×10^4^ spores/mL conidial suspensions of Guy11 were sprayed onto 11-d rice seedlings. Samples were collected at 8, 24, 48 and 72 hpi, respectively. RNA was extracted from all samples using an RNA extraction kit (Invitrogen, USA). The concentration of total RNA was measured and then reverse transcribed (R223-01, Vazyme). Quantitative RT–PCR (qRT–PCR) was performed on a Bio-Rad CFX96 instrument (Bio-Rad, USA) according to the manufacturer’s instructions. The expression of *MoPDX1* at the mycelium stage (MY) was set at 1.0. To evaluate the expression level of *PR* genes, 5×10^4^ spores/mL conidial suspensions of Guy11 and mutant strains were singly sprayed onto 11-d rice seedlings, and samples were collected at 48 hpi. Sterile water sprays were used as negative controls. The stable-expression rice gene *EF1-α* (primers in **Table S1**) was set as an internal control. All experiments were performed for three independent replicates with the same results.

### Co-inmunoprecipitation assays

Hyphal blocks of complementary strains were cultured in liquid CM media at 28°C for 48 h, filtered, and ground into powders in liquid nitrogen as soon as possible. Powders were lysed with 1 mL of protein lysis buffer [10 mM Tris/Cl(pH7.5); 150 mM NaCl; 0.5 mM EDTA; 0.5% NP-40] and 100 mM protease inhibitor PMSF (Cat No. ST506, Beyotime), shaken every 10 min, and then centrifuged at 1,2000 rpm at 4°C for 20 min, the supernatant was used as the total protein. Total protein was co-incubated with commercial GFP beads (Lot 91009001A-04, Ychromotek) at 4°C overnight. Then, the beads were centrifuged at 500 rpm at 4°C for 2 min and washed five times with washing buffer [10 mM Tris/Cl(pH7.5); 150 mM NaCl; 0.5 mM EDTA]. The proteins were eluted within the elution buffer (200 mM glycine, pH2.5), which were neutralized with 1M Tris-base (pH10.4). The candidate interacting proteins of MoPdx1 were further analyzed through mass spectrography (the instrument was LTQ Orbitrap Velos) at Shenzhen BGI Co., LTD, China. The single GFP interaction proteins were used as controls.

### Protein identification analysis

Identified peptides were blasted in the protein database of *M. oryzae* (https://fungidb.org/fungidb/showQuestion.do?questionFullName=UniversalQuestions.UnifiedBlast). The proteins were analyzed in the GO (Gene Ontology), KOG (Eukaryotic orthologous groups), and KEGG (Kyoto Encyclopedia of Genes and Genomes) pathway databases, respectively.

### Statistical analysis

One-way ANOVA analysis with IPM SPSS Statistics Software was used to conduct variance analysis. LSD test was used to make multiple comparisons, the mean difference is significant at the 0.05 level in the whole text. Student-Newman-Keuls test was used to display the means for groups in homogeneous subsets. Three replicates were set for each sample, and all experiments were performed for three independent replicates with the same results.

## Results

### Transcript analysis of *MoPDX1* at different stages of *M. oryzae*

**Figure 1:**
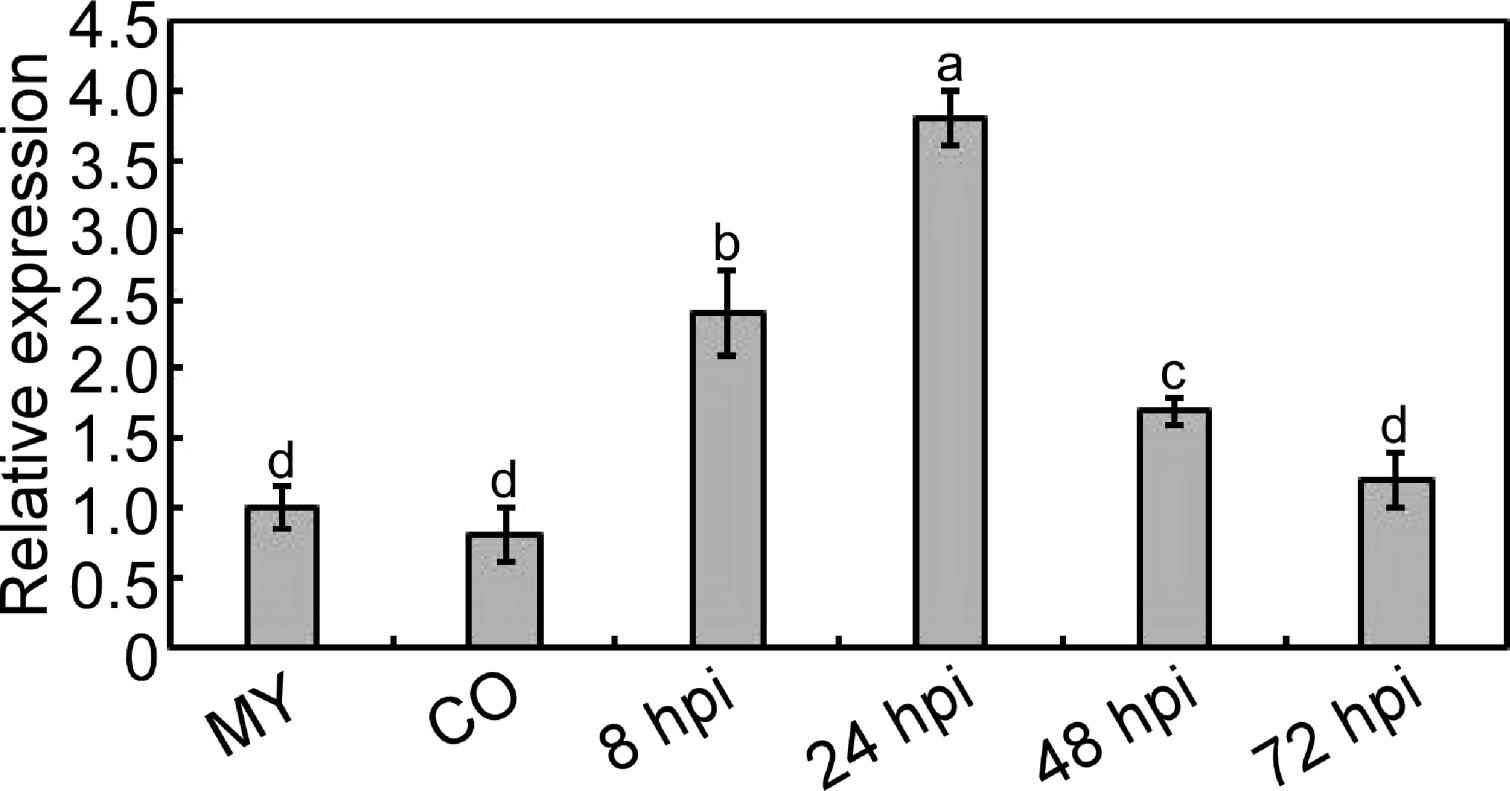
Transcription analysis of *MoPDX1* at different stages of *M. oryzae*. Total RNA was extracted from the mycelium (MY) stages, conidia (CO) stages, and infectious stages at 8, 24, 48, and 72 hours post inoculation (hpi), respectively. The RNA was reverse transcribed and then quantified by q-RT PCR. The transcript level of *MoPDX1* at the mycelium stage was set at 1.0. Experiments were repeated three times with similar results. Error bars represent the standard deviation. Lowercase represent significant differences (LSD and Student-Newman-Keuls test, *p*<0.05).

We identified a gene *MoPDX1* encoded a pyridoxine biosynthesis protein in *M. oryzae*. The transcript level of the gene was analyzed at different stages, such as the mycelium stage (MY), conidial stage (CO), and infectious stage at 8, 24, 48, and 72 hpi. Results showed that the gene *MoPDX1* was significantly upregulated at the early infection stage, approximately 2.4 folds, 3.8 folds, and 1.7 folds at 8, 24, and 48 hpi compared with that of at the mycelium stage, respectively. However, there were no significant differences at the conidial stage and the infectious stage at 72 hpi (**Figure 1**). Suggesting that MoPdx1 might play important roles in the early stage of infection of *M. oryzae*.

### MoPdx1 regulates vegetative growth *via de novo* VB6 biosynthesis in *M. oryzae*

**Figure 2:**
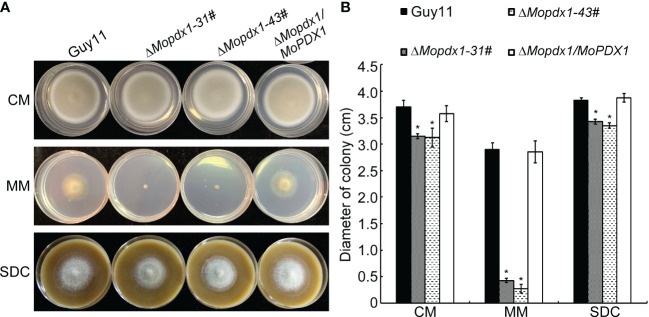
Deletion of *MoPDX1* affects the vegetative growth of *M. oryzae*. **(A)** Colony morphology of Guy11, two *MoPDX1* deletion mutants, and complementary strains after growth at 28°C on CM, MM, and SDC media for 7 days. **(B)** Colony diameters of indicated strains on three kinds of media after 7 days post inoculation (dpi) at 28°C. Experiments were repeated three times with similar results. Error bars represent the standard deviation. Asterisks represent significant differences (LSD and Student-Newman-Keuls test, *p*<0.05).

**Figure 3:**
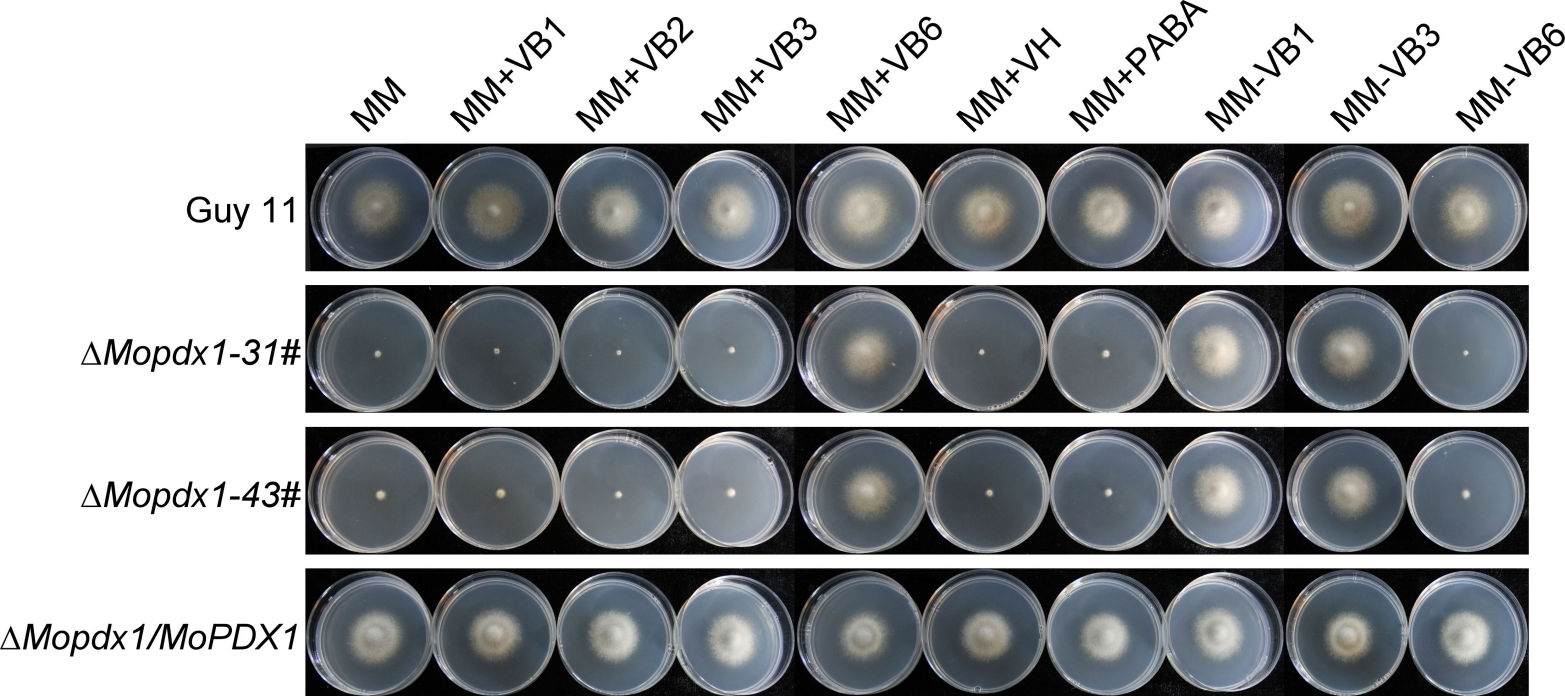
The addition of VB6 but not other kinds of vitamin B rescued the defect in vegetative growth of *MoPDX1* deletion mutants. Hyphal blocks of indicated strains were cultured at 28°C for 7 days in the dark on MM media erther individually supplemented with VB1, VB2, VB3, VB6, VH, or PABA, or only lacking VB1, VB3, and VB6. Experiments were repeated three times with similar results.

To further explore the function of MoPdx1 in *M. oryzae*, the *MoPDX1* gene was replaced with the hygromycin (*HPH*) with the one-step method. Southern blotting confirmed that two transforments were successfully knocked out and that only one copy of *HPH* was inserted into the genome of *M. oryzae* (**Figure S1**). Furthermore, complementary strains were acquired. Vegetative growth of wild-type Guy11, two *MoPDX1* deletion mutants, and complementary strains on CM, MM, and SDC media were measured. The results showed that the vegetative growth of the *MoPDX1* mutants on the three kinds of media all significantly slowed compared to that of Guy11 and complementary strains, especially on MM media, on which the mutants nearly stopped growing (**Figures 2A, B**). Given that MoPdx1 encoding a protein involved in the biosynthesis of pyridoxine, one of VB6 compounds, and there are only basic trace substances in MM media, we want to determine whether vegetative growth defects of the *MoPDX1* mutants could be mitigated by the addition of various vitamin B elements, including VB1, VB2, VB3, VB6, VH, and PABA, which are components of CM media. The results showed that only the exogenous addition of VB6 could entirely rescue the growth defects of two *MoPDX1* deletion mutants, while addition of VB1, VB2, VB3, VH, and PABA individually or together could not (**Figure 3** and **Table 1**). Moreover, the VB6 content in different strains in CM media was measured with HPLC-MS and it was found that there was no significant difference between the WT and *MoPDX1* deletion mutants (**Figure S2** and **Table S2**). Indicating that MoPdx1 regulates the vegetative growth via *de novo* VB6 biosynthesis not in uptake process in *M. oryzae*.

**Table 1:** Statistical analysis of the wild type, Δ*Mopdx1* mutants, and the complement strains on MM media with different types of vitamin B.

Strains	MM (cm)	MM+ VB1 (cm)	MM+ VB2 (cm)	MM+ VB3 (cm)	MM+ VB6 (cm)	MM+ VH (cm)	MM+ PABA (cm)	MM-VB1 (cm)	MM-VB3 (cm)	MM-VB6 (cm)
Guy11	3.7 ± 0.09a	3.8 ± 0.09a	3.9 ± 0.05a	3.7 ± 0.09a	3.9 ± 0.05a	3.8 ± 0.08a	3.6 ± 0.05a	3.8 ± 0.05a	3.7 ± 0.05a	3.6 ± 0.09a
Δ*Mopdx1-31#*	0.6 ± 0.09b	0.7 ± 0.09b	0.5 ± 0.05b	0.7 ± 0.05b	3.8 ± 0.05a	0.5 ± 0.05b	0.5 ± 0.05b	3.7 ± 0.08a	3.7 ± 0.08a	0.6 ± 0.09b
Δ*Mopdx1-43#*	0.6 ± 0.05b	0.6 ± 0.05b	0.5 ± 0.08b	0.6 ± 0.05b	3.7 ± 0.08a	0.4 ± 0.05b	0.5 ± 0.05b	3.7 ± 0.09a	3.7 ± 0.09a	0.5 ± 0.05b
Δ*Mopdx1/MoPDX1*	3.7 ± 0.05a	3.8 ± 0.05a	3.8 ± 0.05a	3.7 ± 0.05a	3.7 ± 0.05a	3.7 ± 0.05a	3.5 ± 0.05a	3.6 ± 0.05a	3.6 ± 0.05a	3.7 ± 0.05a

± SD was calculated from three repeated experiments and lowercase indicates statistically significant differences (LSD and Student-Newman-Keuls test, p<0.05).

### Deletion of *MoPDX1* affects the appressorium turgor pressure of *M. oryzae*

We further evaluated conidiation, conidial germination, appressorium formation, and turgor pressure in Guy11, two *MoPDX1* deletion mutants, and their complementary strains. The results showed that there was no significant difference in conidiation, conidial germination, or appressorium formation between Guy11 and the deletion mutants (**Table 2**). However, appressorium collapse rate in the two *MoPDX1* deletion mutants rose dramatically at 2-4 M glycerol compared with that of in Guy11 and complementary strains, approximately 64.8% under the treatment of 2 M glycerol, 84.2% under the treatment of 3 M glycerol and 91.9% under the treatment of 4 M glycerol; while it was significantly reduced in the deletion mutants at 1 M glycerol (**Table 2**). The results indicate that the deletion of *MoPDX1* affects the appressorium turgor pressure of *M. oryzae.*

**Table 2:** Conidiation, conidial germination, appressorium formation, and turgor pressure assays of the wild type, Δ*Mopdx1* mutant, and the complement strains.

Strains	Conidiation (x100/cm^2^)^I^	Conidial germination (%)^II^	Appressorium formation (%)^III^	Appressorium exhibiting collapse rate (%)^IV^			
				1 M	2 M	3M	4 M
Guy11	559.8 ± 9.4a	99.7 ± 0.6 a	98.5 ± 1.2a	21.3 ± 2.0a	52.4 ± 2.8b	62.7 ± 4.1c	77.9 ± 2.6b
Δ*Mopdx1-31#*	556.6 ± 56.7a	99.3 ± 1.0 a	99.0 ± 0.4a	9.1 ± 2.2b	63.2 ± 1.6a	79.6 ± 1.6b	91.7 ± 1.6a
Δ*Mopdx1-43#*	565.6 ± 43.4a	99.0 ± 1.1 a	98.7 ± 1.6a	10.4 ± 0.6b	66.3 ± 4.4a	88.8 ± 2.0a	92.1 ± 1.2a
Δ*Mopdx1/MoPDX1*	530.6 ± 24.3a	99.0 ± 1.0 a	99.0 ± 0.9a	21.6 ± 1.6a	51.0 ± 2.2b	65.3 ± 1.2c	79.4 ± 1.4b

I. Hyphal blocks of different strains were cultured on SDS media in the dark for 7 days, then removed at continuous illumination under fluorescence light for another 3 days. Conidia were collected through a filter by the close filter and measured the conidiation. ± SD was calculated from three repeated experiments and lowercase indicates statistically significant differences (LSD and Student-Newman-Keuls test, p<0.05).

II. Conidia were collected through a filter by the close filter, then 20 μL of conidial suspensions of different strains were put on hydrophobic surfaces. Conidiation germination was measured at 6 hours post incubation (hpi). At least 100 conidia were counted. ± SD was calculated from three repeated experiments and lowercase indicates statistically significant differences (LSD and Student-Newman-Keuls test, p<0.05).

III. Appressorium formation on hydrophobic surfaces at 24 hpi. At least 100 appressoria were counted. ± SD was calculated from three repeated experiments and lowercase indicates statistically significant differences (LSD and Student-Newman-Keuls test, p<0.05).

IV. Different concentrations of glycerol (1-4 M) to analyze incipient cytorrhysis. At least 100 appressoria were counted for each concentration. ± SD was calculated from three repeated experiments and lowercase indicates statistically significant differences (LSD and Student-Newman-Keuls test, p<0.05).

### MoPdx1 is involved in the pathogenicity of *M. oryzae*

**Figure 4:**
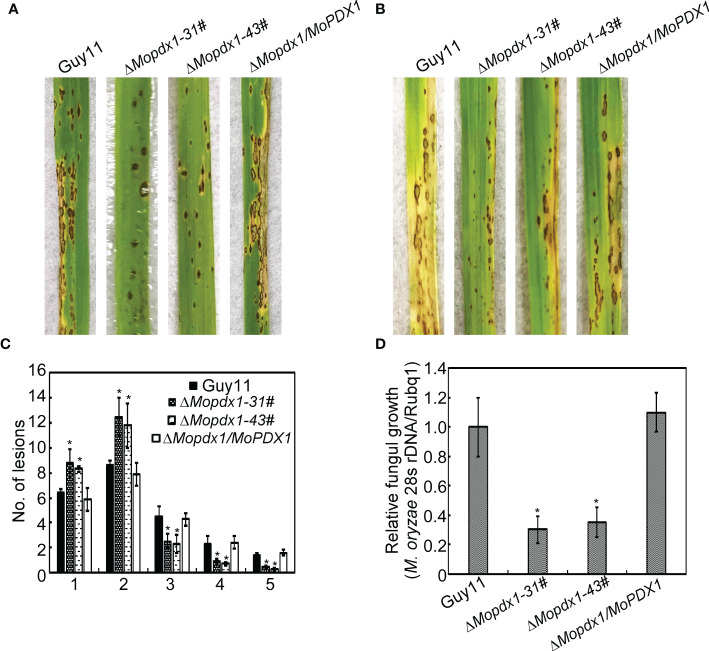
MoPdx1 regulates the pathogenicity of *M. oryzae*. **(A)** Five milliliters of conidial suspensions (5×10^4^ spores/mL) of the indicated strains were severally sprayed onto 11-d rice CO-39 seedlings in a moist chamber with 90% relative humidity at 28°C. The diseased leaves were photographed at 7 dpi. **(B)** Conidial suspensions (15×10^4^ spores/mL) were injected into 21-d rice sheath cells. Photographs were taken at 5 dpi. **(C)** Lesion-type statistical analysis at 7 dpi. Lesion-type classifications were as referred to Yang et al., 2019. Error bars represent the standard deviation. Asterisks represent significant differences (LSD and Student-Newman-Keuls test, *p*<0.05). **(D)** Relative fungal content analysis in leaves of equal area by quantification of *M. oryzae* 28S rDNA relative to rice Rubq1. Error bars represent the standard deviation. Asterisks represent significant differences (LSD and Student-Newman-Keuls test, *p*<0.05).

The appressorium is an important structure of *M. oryzae* which produces penetrating pegs that successfully penetrate into the host cell. Deletion of *MoPDX1* affected the appressorium turgor of *M. oryzae*; therefore, virulence assays were performed. The 5×10^4^ spores/mL of conidial suspensions of Guy11, two *MoPDX1* deletion mutants and complementary strains were sprayed on 11-d rice CO-39 seedlings, and disease symptoms were observed at 7 days post inoculation (dpi). The two *MoPDX1* deletion mutants presented the most restricted lessons and bits of typical lesions of rice blast compared to that caused by Guy11 and complementary strains (**Figures 4A, C**). In addition, lesion biomass assays of all the diseased leaves suggested that the content of *M. oryzae* was markedly reduced treated with the two Δ*Mopdx1* mutants (**Figure 4D**). Moreover, conidial suspensions (15×10^4^ spores/mL) of the different strains was injected into 21-d rice sheath cells and observed at 5 dpi. Lesion incidence was in keeping with that of rice spraying, with lesions reduced by two-thirds in the two Δ*Mopdx1* mutants (**Figure 4B**). Overall, the results suggested that MoPdx1 is involved in the pathogenicity of *M. oryzae*.

### *De novo* VB6 biosynthesis plays an important role in the invasive growth of *M. oryzae*

**Figure 5:**
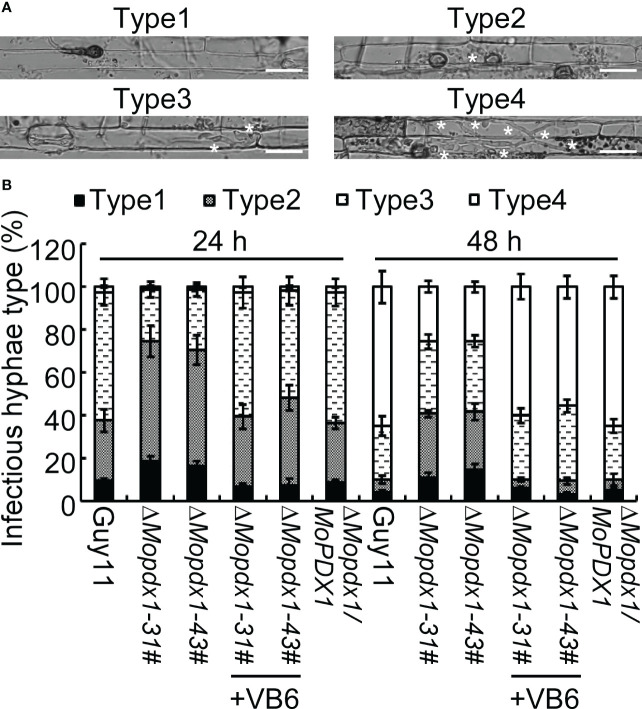
Deletion of *MoPDX1* attenuates the growth of infectious hyphae in rice sheath cells. **(A)** Four types of infectious hyphae (IH) of *M. oryzae* in rice sheath cells. Type1 has no penetration; type 2 only has penetration peg or a single infectious hypha (IH); type 3 has more than two IH in one rice cell; type 4 has extensive IH into adjacent rice cells. White asterisks indicate IH in rice sheath cells. **(B)** Statistical analysis of the growth of IH in rice cells at 24 and 48 hpi with or without 10^-4^ mg/mL of VB6 treating the conidial suspensions of *MoPDX1* deletion mutants. One hundred IH were counted in rice cells infected with conidia suspensions of the indicated strains. Error bars represent the standard deviation. The experiments were repeated three times with similar results.

To further explore the causes of the reduced pathogenicity of the two Δ*Mopdx1* mutants, the growth of infectious hyphae in 21-d rice sheath cells injected with conidial suspensions of Guy11, two *MoPDX1* deletion mutants, or the complementary strains were observed at 24 and 48 hpi, respectively. Four types of invasive hyphae are shown in **Figure 5A**. In Guy11 and complementary strains, type 1 invasive hyphae accounted for 8%, 30% of cells were type 2, 60% of cells were type 3 and 2% of cells were type 4 at 24 hpi. In the two Δ*Mopdx1* mutants, 18% of cells were type 1, 56% of cells were type 2 and 24% were type 3 (**Figure 5B**). Most of the cells (approximately 65%) were type 4 and 25% of cells were type 3 in Guy11 and complementary strains at 48 hpi. However, in the two mutant strains, 30% were still type 2, 34% were type 3, and 26% were type 4 (**Figure 5B**). In addition, conidial suspensions of *MoPDX1* deletion mutants were added 10^-4^ mg/mL of VB6 solutions, and then injected into the rice shealth cells, observed the invasive growth at 24 and 48 hpi. Results found that addition of VB6 could rescue the defects of invasive growth of the mutants whether inoculated 24 or 48 h (**Figure 5B**). The results suggest that *de novo* VB6 plays an important role in invasive growth of *M. oryzae*.

### Exogenous addition of VB6 rescue the pathogenicity defect of the Δ*Mopdx1* mutants

**Figure 6:**
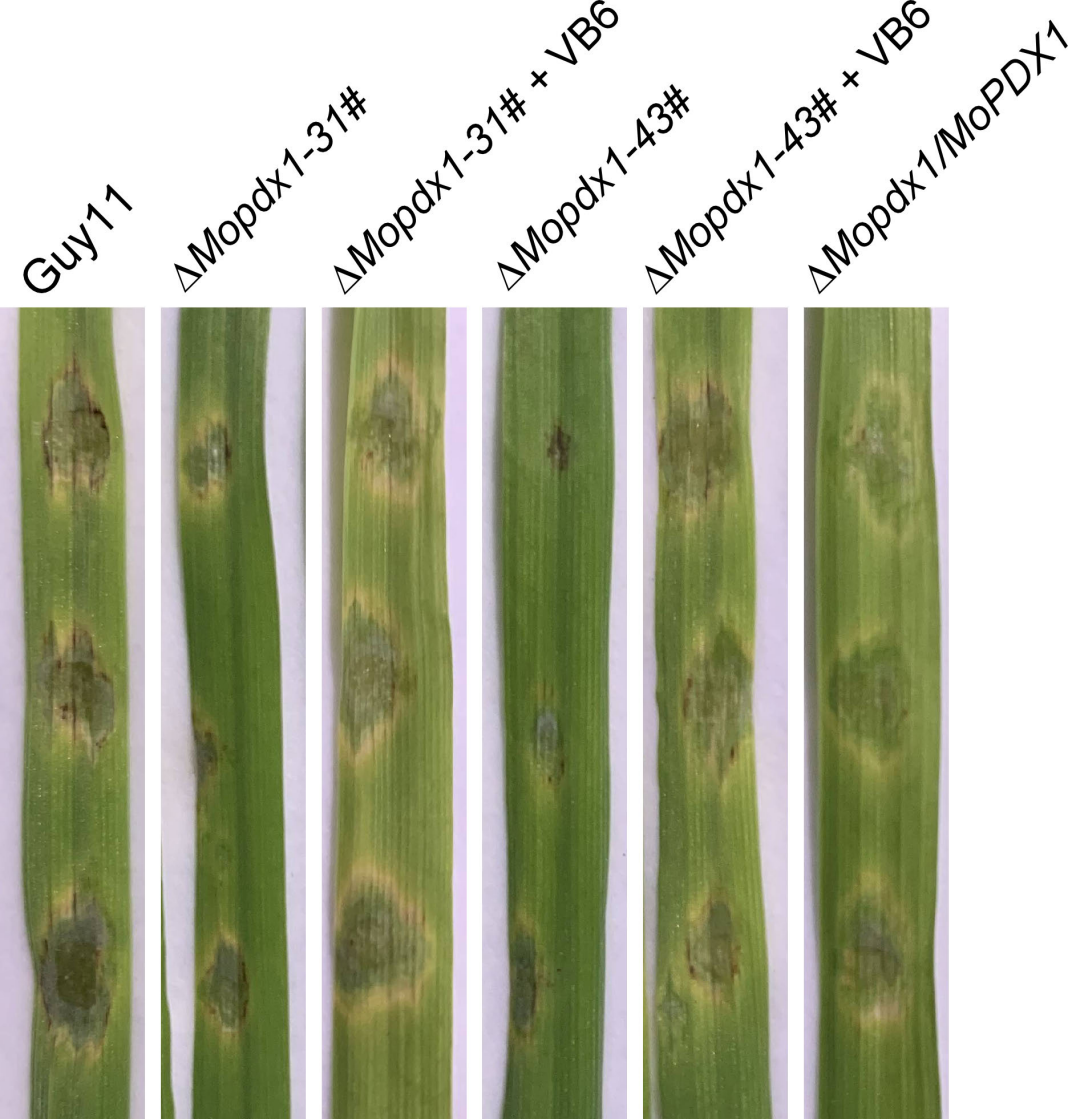
The addition of VB6 rescues the pathogenicity defect of *MoPDX1* deletion mutants on barely leaves. Twenty-five microliters of conidial suspensions (5×10^4^ spores/mL) of Guy11, two Δ*Mopdx1* mutants, and the complementary strains were separately inoculated on the surface of 7-d detached barely leaves, which were then inoculated at 28°C for 24 h in the dark, and for another 4 days under continuous12 h/12 h light/dark conditions. In addition, conidial suspensions of the two mutants were added 10^-4^ mg/mL VB6 solution. Then, inoculated on the barely leaves under the same environmental conditions as described above. The experiments were repeated three times with similar results.

Further, conidial suspensions of *MoPDX1* deletion mutants with 10^-4^ mg/mL VB6 solutions were then inoculated onto the detached 7-d barely leaves, and the lesion incidence was observed at 5 dpi. Results showed that the exogenous addition of commercial VB6 could also rescue the pathogenicity defect of the two Δ*Mopdx1* mutants on barely leaves (**Figure 6**). Indicating that VB6 is involved in the virulence of *M. oryzae*.

### Deletion of *MoPDX1* does not activate the defense response of rice

**Figure 7:**
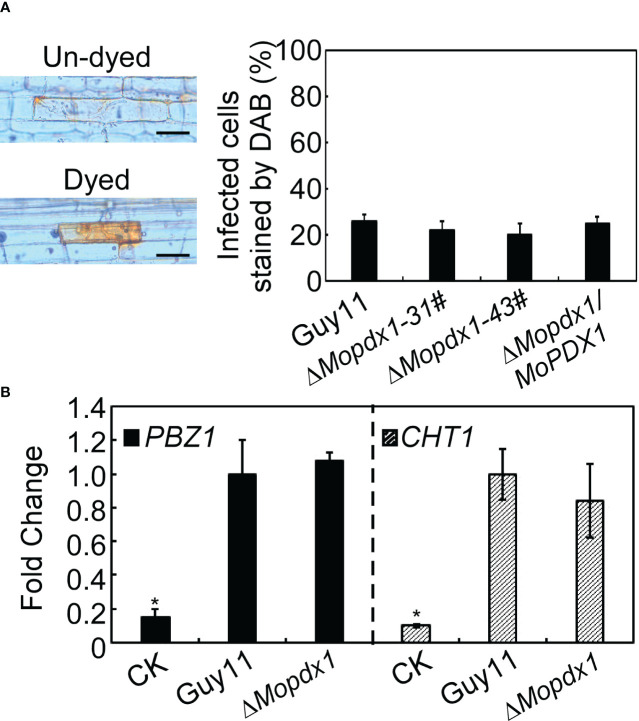
Deletion of *MoPDX1* does not activate the defense response of rice. **(A)** Conidia suspensions (15×10^4^ spores/mL) of indicated strains were injected into 21-d rice sheath cells, DAB (3,3’-diaminobenzidine) was used to dye the ROS produced in rice sheath cells for 10 h at 24 hpi, and then decolorated by alcohol and acetic acid for 4 h. The left panel showed dyed or un-dyed representative photos. The right panel showed the statistical analysis. Error bars represent the standard deviation. There was no significant difference under the different treatments in indicated strains (LSD and Student-Newman-Keuls test, *p*<0.05). **(B)** Transcription analysis of the rice defense-related genes *PBZ1* and *CHT1* by qRT-PCR. Conidial suspensions (5×10^4^ spores/mL) of Guy11 and Δ*Mopdx1* mutants were separately sprayed onto 11-d rice CO-39 seedlings, rice leaves were collected at 48 hpi and RNA was extracted. Sterile water treatment was used as a control (CK). The stablely expressed rice gene *EF1-α* was used as an internal control. Error bars represent the standard deviation. Asterisks represent significant differences (LSD and Student-Newman-Keuls test, *p*<0.05). All experiments were performed for three independent replicates with the same results.

In general, plants produce a broad-spectrum defense response against the attack of pathogens, including rapid generation of ROS, and changes in the expression levels of pathogenesis-related (PR) proteins (Bradley et al., 1992; Tanaka et al., 2003). We want to determine whether the reduced pathogenicity of the Δ*Mopdx1* mutants was due to defense responses activated by the host. 3,3’- diaminobenzidine (DAB) was used to stain the ROS in rice sheath cells after inoculation for 48 h. The results showed that the ROS level was not significantly different caused by WT and the two Δ*Mopdx1* mutants, and approximately 25% of rice sheath cells were stained with DAB (**Figure 7A**). In addition, the transcript levels of rice defense-related genes *PBZ1* and *CHT1* (Chen et al., 2014; Liu et al., 2016b) were measured in the rice leaves infected for 48 h by spores of Guy11 and Δ*Mopdx1* mutants, separately. Water-sprayed rice leaves were used as negative controls. The transcript levels of *PBZ1* and *CHT1* were also not significantly different between Guy11 and Δ*Mopdx1* mutants (**Figure 7B**). Results illustrated that deletion of *MoPDX1* does not activate plant defense responses.

### MoPdx1 is located in the cytoplasm of *M. oryzae*

**Figure 8:**
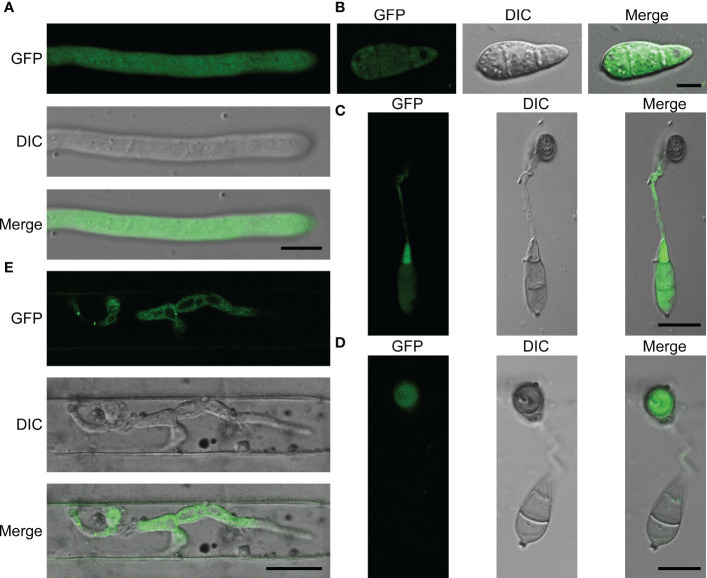
MoPdx1 is located in the cytoplasm of *M. oryzae.* **(A)** Hyphal blocks of complementary strains were cultured in liquid CM media at 28°C for 18 h, washed two times with sterile water, and single mycelium were photographed under a confocal fluorescence microscope (Zeiss LSM710, 63x oil). Bar=18 μm. **(B)** Conidia of complementary strains were observed under a confocal fluorescence microscope (Zeiss LSM710, 63x oil). Bar=18 μm. **(C, D)** Appressorium formation was observed under a confocal fluorescence microscope (Zeiss LSM710, 63x oil) at 14 and 24 hpi. Bar=18 μm. **(E)** Conidial suspensions (5×10^4^ spores/mL) of the complementary strains were collected and inoculated into 21-d rice sheath cells, and the green fluorescence of IH were observed at 24 hpi under a confocal fluorescence microscope (Zeiss LSM710, 63x oil). Bar=18 μm.

MoPdx1 co-expressed with a GFP protein to detect its localization at different development stages of *M. oryzae*, including the mecelium stage, infectious hyphal stages, conidial stage, and appressorium formation stage. The results showed that MoPdx1 was located in the cytoplasm at the mecelium stage, conidial stage, and infectious hyphal stages (**Figures 8A, B, E**). Furthermore, MoPdx1 was present in spore and germ tubes at 14 hpi and then transferred into the appressorium at 24 hpi (**Figures 8C, D**). Suggesting that MoPdx1 is located in the cytoplasm of *M. oryzae*.

### MoPdx1 is involved in multiple pathways of *M. oryzae*

**Figure 9:**
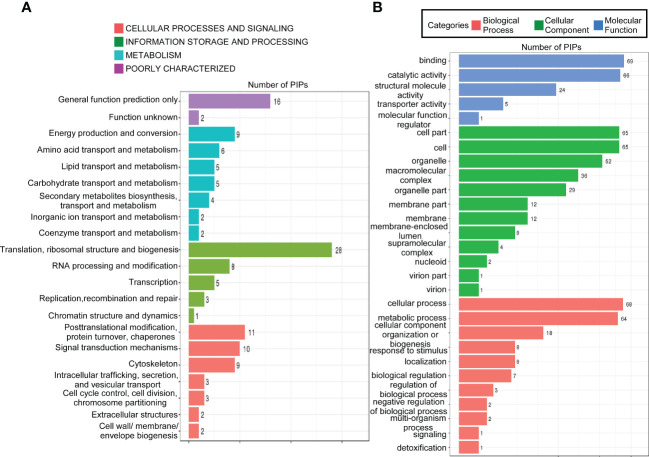
Functional analysis of potential interacting proteins of MoPdx1 in *M. oryzae*. **(A)** KOG analysis of potential interaction proteins (PIPs) of MoPdx1. The Y-axis represents the analysis categories, including cellular processes and signaling, information storage and processing, metabolism, and poorly characterized. The X-axis represents the number of PIPs. **(B)** GO analysis of PIPs of MoPdx1. The Y-axis represents the analysis categories, including biological process, molecular function, and cellular component. The X-axis represents the number of PIPs.

To gain further insight into the function of MoPdx1 in *M. oryzae*, its interacting proteins were enriched by GFP beads using two strains of Guy11 expressing MoPdx1-GFP. A Guy11 strain with only GFP was used as a control. The results showed that 10 proteins had more than two peptides that potentially interacted with the MoPdx1 protein in both two MoPdx1-GFP expressing strains, except the proteins in only GFP expressing strain (**Table 3**). Furthermore, two proteins previously studied by our laboratory encoded by MGG_11513 (Yang et al., 2018) and MGG_04719 (Yin et al., 2018) were selected to identify the interaction with MoPdx1 through yeast two-hybrid (Y2H), respectively. Both proteins could physically interact with MoPdx1 on -Trp/-Leu/-His medium under 5 mM 3-AT (**Figure S3**), conforming the reliability of the Co-IP results. In this work, 146 proteins and 275 peptides were identified also in both two MoPdx1-GFP expressing strains, except the proteins in only GFP expressing strain, and further analyzed the function of these IP proteins, which might elucidate the function of MoPdx1 in *M. oryzae*. Eukaryotic Orthologous Groups (KOG) is a database for identifying orthologous and paralogous proteins. KOG database showed the IP proteins of MoPdx1 mainly focused on translation, ribosomal structure and biogenesis, general function prediction only, posttranslational modification, protein turnover, chaperones, signal transduction mechanisms, cytoskeleton, energy production and conversion, and RNA processing and modification (**Figure 9A**). The top 10 GO enrichment terms were binding, cellular process, catalytic activity, cell part, cell, metabolic process, organelle, macromolecular complex, organelle part, and structural molecular activity (**Figure 9B**). The top 10 pathways from KEGG pathway enrichment analysis were metabolic pathways, ribosome, biosynthesis of antibiotics, biosynthesis of secondary metabolites, biosynthesis of amino acids, carbon metabolism, protein processing in the endoplasmic reticulum, spliceosome, pyruvate metabolism, and endocytosis (**Table 4**). Therefore, MoPdx1 takes part in multiple pathways of *M. oryzae*, especially in the ribosome and in biosynthesis of various substances.

**Table 3:** Co-IP assays identified more than two peptides that potentially interacted with the MoPdx1 protein in both two MoPdx1-GFP expressing strains, except the proteins in only GFP expressing strains.

Gene symbol	Protein_ID	Protein _Qscore	Unique_ Peptide_ Num	Coverage	Description
MGG_05980	XP_003711825.1	69.6144997148	16	0.4389	Pyridoxine biosynthesis protein PDX1
MGG_01742	XP_003714739.1	39.4505104539	12	0.1386	elongation factor 2
MGG_06712	XP_003709423.1	29.9177503462	10	0.1345	5-methyltetrahydropteroyltriglutamate-homocysteine S-methyltransferase
MGG_11513	XP_003718330.1	22.1969984131	6	0.0961	heat shock protein SSB1
MGG_04719	XP_003710816.1	12.728009214	5	0.1361	guanine nucleotide-binding protein subunit beta-like protein
MGG_12805	XP_003717014.1	13.0879956837	3	0.0474	tryptophan synthase
MGG_05193	XP_003712735.1	9.86281428633	2	0.0268	cell division control protein 48
MGG_00444	XP_003718624.1	8.46983625667	2	0.089	uncharacterized protein MGG_00444
MGG_10357	XP_003719394.1	5.34060745338	2	0.0406	prolyl-tRNA synthetase
MGG_06650	XP_003709356.1	5.14729053786	2	0.0667	tubulin alpha-B chain
MGG_15774	XP_003714819.1	7.73323381058	2	0.0525	ketol-acid reductoisomerase

**Table 4:** Top 10 pathway from KEGG pathways functional enrichment analysis.

Pathway	Proteins with pathway annotation (118)	Pathway ID	Level 1	Level 2
Metabolic pathways	24 (20.34%)	ko01100	Metabolism	Global and overview maps
Ribosome	22 (18.64%)	ko03010	Genetic Information Processing	Translation
Biosynthesis of antibiotics	16 (13.56%)	ko01130	Metabolism	Global and overview maps
Biosynthesis of secondary metabolites	15 (12.71%)	ko01110	Metabolism	Global and overview maps
Biosynthesis of amino acids	10 (8.47%)	ko01230	Metabolism	Global and overview maps
Carbon metabolism	8 (6.78%)	ko01200	Metabolism	Global and overview maps
Protein processing in the endoplasmic reticulum	5 (4.24%)	ko04141	Genetic Information Processing	Folding, sorting, and degradation
Spliceosome	5 (4.24%)	ko03040	Genetic Information Processing	Transcription
Pyruvate metabolism	5 (4.24%)	ko00620	Metabolism	Carbohydrate metabolism
Endocytosis	4(3.39%)	ko04144	Cellular Processes	Transport and catabolism

## Discussion

In this study, we identified the pyridoxine biosynthesis protein Pdx1 of *M. oryzae*. Deletion of *MoPDX1* made it almost impossible to grow on MM media, exogenous addition of VB6 alone or VB6-containing compounds entirely rescued the growth defect of the mutants on MM media. VB6 content detection showed that there was no significant difference in Guy11, *MoPDX1* deletion mutants and complementary strains cultured in liquid CM media. Suggesting that the MoPdx1 protein is involved in *de novo* VB6 biosynthes not in uptake process, and VB6 plays an important role in the vegetative growth of *M. oryzae*. However, the vegetative growth of the mutants still has significant difference compared with Guy11 and complementary strains in CM media, indicated that there still had other factors (such as MoPdx1 interacting protein) affect the vegetative growth of the *MoPDX1* deletion mutants. The Δ*Mopdx1* mutants affected appressorium turgor but not affect appressorium formation, indicating that MoPdx1 might take part in the penetrating process of *M. oryzae*. Besides that, MoPdx1-GFP signals were detected in conidia and germ tube at 14 hpi, further found that the GFP signals were detected in appressoria at 24 hpi in **Figures 8C, D**. Further, appressorium turgor pressure in the wild type and *MoPDX1* deletion mutants were detected at 14 and 24 hpi, found that the appressorium turgor pressure in the mutants were significantly decreased at 2-4 M glycerol compared with that of in wild type at both 14 and 24 hpi. Addition of VB6 in the conidial suspensions at the *MoPDX1* deletion mutants could rescue the defects of appressorium turgor pressure at both 14 hpi and 24 hpi (**Table 5**). The maturation of appressorium of *M. oryzae* is accompanied by degradation of substances in spores and then transferring to appressorium (Davis et al., 2000; Veneault-Fourrey et al., 2006; Wang et al., 2007; Foster et al., 2017). Therefore, MoPdx1 affected the appressorium function mainly depended on VB6, and VB6 was biosynthesized in conidia and then transported into the appressoria. It is possible that the main function of VB6 was in the transfer of substances from conidia to appressorium at the early stage of appressorium formation (14 hpi), which is crucial for appressorium turgor pressure. Furthermore, deletion of *MoPDX1* observably attenuated pathogenicity in rice seedlings and sheaths. The results of the growth of infectious hyphae assay at 24 hpi and 48 hpi, respectively, suggested that attenuated pathogenicity in the Δ*Mopdx1* mutants was due to the slower growth rate of infectious hyphae. Addition of VB6 in the conidial suspensions of the mutants rescued the invasive growth defects whether inoculated 24 or 48 h. And continuous exogenous addition of VB6 rescued the pathogenicity defect of the mutants on barely leaves. All these results demonstrated that VB6 plays important roles in the development and virulence of *M. oryzae* regulating vegetative growth, appressorium turgor pressure and invasive growth. In *M. oryzae*, essential roles in development and pathogenicity were play by amino acid and nutrient metabolism, including methionine metabolism (MoStr3, MoMet6, MoMet13) (Wilson et al., 2012; Yan et al., 2013; Saint-Macary et al., 2015), arginine biosynthesis (MoArg1, MoArg5,6, MoArg7, MoCpa2) (Zhang et al., 2015; Liu et al., 2016a), leucine, isoleucine and valine biosynthesis (MoIlv1, MoIlv2, MoIlv6) (Du et al., 2013; Du et al., 2014), and purine metabolism (MoAde1, MoImd4, MoAde12) (Fernandez et al., 2013; Yang et al., 2019; Zhang et al., 2022). In addition, *M. oryzae* could not effectively utilize VB6 from rice cells, and must synthesize it *in vivo* for successful invasion.

**Table 5:** Appressorium turgor pressure assays of the wild type, Δ*Mopdx1* mutant, and the complement strains with or without 10^-4^ mg/mL of VB6 at 14 or 24 hpi, respectively.

strains	Appressorium collapse rate (%)															
	14 hpi								24 hpi							
	-VB6				+VB6				-VB6				+VB6			
	1 M	2M	3M	4M	1 M	2 M	3 M	4 M	1 M	2 M	3 M	4 M	1 M	2 M	3 M	4 M
Guy11	18.7 ± 2.5a	63.3 ± 5.5b	78.7 ± 7.6b	85.3 ± 1.5b	19.0 ± 3.6b	62.0 ± 3.6b	77.0 ± 4.5b	84.0 ± 1.0b	19.3 ± 1.5a	52.3 ± 3.8b	62.7 ± 1.5b	80.7 ± 7.5b	21.7 ± 3.5b	54.0 ± 5.3b	62.0 ± 2.6b	78.0 ± 6.1b
Δ*Mopdx1-31#*	7.0 ± 2.0b	73.7 ± 5.5a	88.0 ± 3.0a	94.0 ± 3.6a	18.0 ± 2.0b	61.7 ± 5.5b	79.7 ± 4.5b	85.7 ± 0.6b	7.3± 1.5b	64.0 ± 2.6a	76.0 ± 6.0a	90.0 ± 3.0a	19.0 ± 1.0b	54.7 ± 3.1b	63.0 ± 1.0b	76.0 ± 3.6b
Δ*Mopdx1-43#*	9.7 ± 3.2b	72.7 ± 4.1a	87.3 ± 4.5a	94.7 ± 1.5a	21.0 ± 2.0b	62.3 ± 2.1b	77.3 ± 2.1b	83.0 ± 2.0b	8.0 ± 5.2b	63.7 ± 3.8a	79.7 ± 1.5a	91.7 ± 1.5a	18.7 ± 3.2b	43.7 ± 4.0b	63.7 ± 3.8b	82.0 ± 2.6b
Δ*Mopdx1/MoPDX1*	20.0 ± 2.0b	63.0 ± 7.8b	75.7 ± 3.5b	84.3 ± 2.5b	20.3 ± 2.5b	63.7 ± 3.2b	76.0 ± 2.0b	84.7 ± 0.6b	20.7 ± 2.5a	51.0 ± 2.0b	63.3 ± 1.5b	81.0 ± 1.0b	20.3 ± 2.5b	57.0 ± 3.0b	62.7 ± 2.1b	81.7 ± 2.5b

Concentrations of 1-4 M of glycerol were used to analyze incipient cytorrhysis with or without 10-4 mg/mL of VB6 treatments. At least 100 appressoria were counted for each concentration. ±SD was calculated from three repeated experiments and lowercase indicates statistically significant differences (LSD and Student-Newman-Keuls test, p<0.05).

In general, conserved pathogen-associated molecular patterns (PAMPs) triggering plant immunity are the first layer of defense against pathogens with the production of ROS. In the long course of evolution, ROS scavenging system produced by pathogens has helped them successfully invade the host and bypass plant immunity (Nathues et al., 2004; Chi et al., 2009). In this study, DAB staining assays were used to evaluate the ROS content in rice sheath cells, and no significant difference was found in the ROS accumulation in rice cells treated with conidial suspensions of either wild-type or *MoPDX1* deletion mutants. Furthermore, the transcript levels of defense-related genes (*PBZ1* and *CHT1*) showed no significant difference, suggested that deletion of *MoPDX1* didn’t activate host defense responses. Similar results were previously found for Δ*Moade1*, Δ*Mocpa2*, and Δ*Moimd4* mutants (Fernandez et al., 2013; Liu et al., 2016b; Yang et al., 2019).

In this work, we also explored potential interacting proteins of MoPdx1 by Co-IP assays *in vivo* to further determine its function in *M. oryzae*. Ten proteins presented more than two peptides in both MoPdx1-GFP expressing strains except the proteins in only GFP expressing strains. Two proteins of them (encoded by MGG_04719 and MGG_11513) were confirmed to interact with MoPdx1 through the Y2H system, suggesting the reliability of the Co-IP results. MoMip11 protein encoded by MGG_04719 has been reported that linked with MoRgs7 and G-proteins to regulate cAMP signaling, stress responses and pathogenicity of *M. oryzae* (Yin et al., 2018). The heat-shock protein MoSsb1 (encoded by MGG_11513), which forms a complex with MoSsz1 and MoZuo1, regulated the growth, conidiation, and pathogenicity of *M. oryzae* mediating cell wall integrity signaling pathway by modulating MoMkk1 biosynthesis (Yang et al., 2018). MoMet6 (encoded by MGG_06712) was a cobalamine-independent methionine synthase, and deletion of *MoMET6* lead to nonpathogenic effects on both barely and rice leaves, defective appressorium-mediated penetration, infectious hyphal growth, abnormal vegetative growth, and metabolic perturbations of *M. oryzae* (Saint-Macary et al., 2015). Mutations of the homologous genes of MGG_10357 and MGG_15774-encoded proteins were inviable in *Saccharomyces*, showing that these proteins might play essential roles in the development of organisms. In addition, the elongation factor has been reported as a virulence pathogenic factor of pathogens (Hwang et al., 2013; Nouripour-Sisakht et al., 2017; Cui et al., 2018). Tubulin is an important component of the cytoskeleton of organisms. The importance of these potentially interacting proteins at the development and pathogenicity stages suggests that MoPdx1 has an important regulatory effect on the development and virulence of *M. oryzae*. In addition, the bioinformatic analysis showed that MoPdx1 might participate in multiple pathways, especially in the ribosome and in the biosynthesis of various substances.

However, there were still some inconsistencies between this work and other studies. We found that MoPdx1 was located in the cytoplasm at the hyphal stage, infectious hyphal stages, and conidial stage of *M. oryzae*. However, previous reports found that Pdx1 in *C. nicotianae* was located in vesicles (Chung et al., 2002). In addition, MoPdx1 was not found in this study to be involved in the oxidative (mainly H_2_O_2_) stress response of *M. oryzae* (**Figure S4**). A previous study indicated that Pdx1 in *Arabidopsis*, tomatoes, and *C. nicotianae* might be an antioxidant (Ehrenshaft et al., 1999; Chen and Xiong, 2005; Zhang et al., 2014). The reason for these differences might be the difference in species.

## Data availability statement

The original contributions presented in the study are included in the article/**Supplementary Material**. Further inquiries can be directed to the corresponding author.

## Author contributions

LY, LL, WF, and ZJ designed and supervised experiments and wrote the manuscript. XL and JW performed the experiments and analyzed the data. All authors contributed to the article and approved the submitted version.

## Supplementary material

Supplementary Figure 1 Southern blot analysis of the *MoPDX1* deletion mutants. **(A)** The strategy of *MoPDX1* disruption. The coding region of *MoPDX1* was replaced with a hygromycin phosphotransferase (*HPH*) fragment. **(B)** Southern blot analysis of two *MoPDX1* deletion mutants with a specific inner probe (probe 1) and *HPH* probe (probe 2).

Supplementary Figure 2 Measurement of the intracellular VB6 content of different strains on CM media by HPLC-MS analysis. **(A-D)** HPLC-MS spectra for detecting the intracellular VB6 content of the indicated strains. The peak flow of the VB6 standard was at ~1.25 min.

Supplementary Figure 3 Interaction of MoPdx1 with proteins encoded by MGG_11513 and MGG_04719 in a yeast two hybrid assay, respectively. *MoPDX1* was inserted into vector pGADT7, and MGG_11513 or MGG_04719 were individually inserted into vector pGBKT7. The latter were co-transferred separately with the *MoPDX1* vector into yeast AH109 cells and incubated on SD-Leu-Trp for 3 days, then selected on SD-Leu-Trp-His with 1 mM X-α-gal and 5 mM 3-AT (3-amino-1,2,4-triazole) for another 3 days.

Supplementary Figure 4 MoPdx1 is not involved in the oxidative stress response of *M. oryzae*. Hyphal blocks of the indicated strains were cultured on CM media with or without 2.5 or 5.0 mM H_2_O_2_ at 28°C in the dark. The colony diameters were measured at 7 dpi. Error bars represent the standard deviation. There was no significant difference under the different treatments in indicated strains (LSD and Student-Newman-Keuls test, *p*<0.05). All experiments were performed for three independent replicates with the same results.

Supplementary Table 1 Primers used in this study.

Supplementary Table 2 Measurement of the content of VB6 in different strains on CM media by HPLC-MS.
