# Supplementary figures and images for: Pyridoxine biosynthesis protein MoPdx1 affects the development and pathogenicity of Magnaporthe oryzae
Source: Front Cell Infect Microbiol. 2023 Feb 7;13:1099967. doi: 10.3389/fcimb.2023.1099967 (PMC9941553; doi:10.3389/fcimb.2023.1099967)

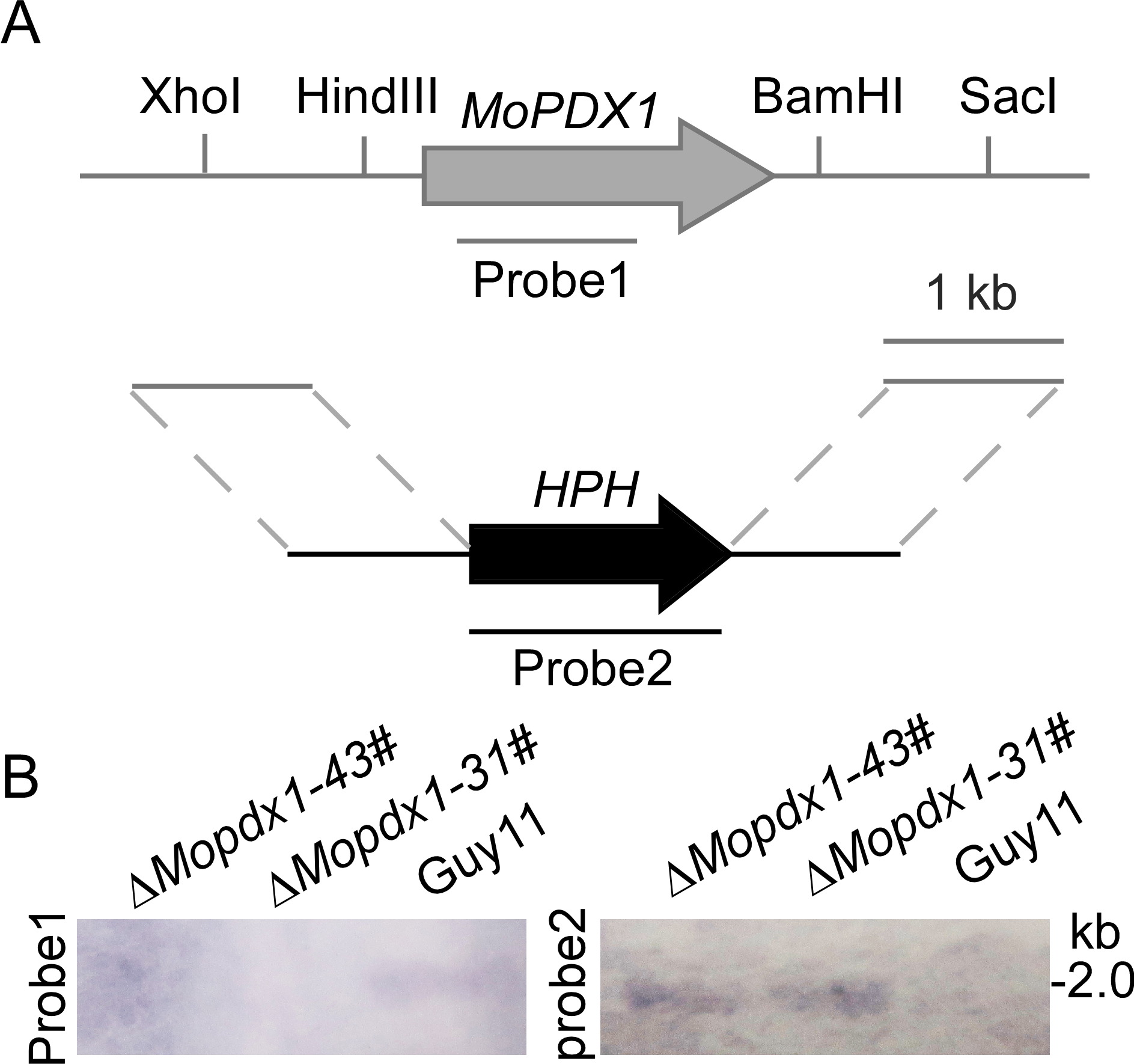

Supplement: Supplementary Figure 1 — Southern blot analysis of the MoPDX1 deletion mutants. (A) The strategy of MoPDX1 disruption. The coding region of MoPDX1 was replaced with a hygromycin phosphotransferase (HPH) fragment. (B) Southern blot analysis of two MoPDX1 deletion mutants with a specific inner probe (probe 1) and HPH probe (probe 2). [file Image_1.tif]

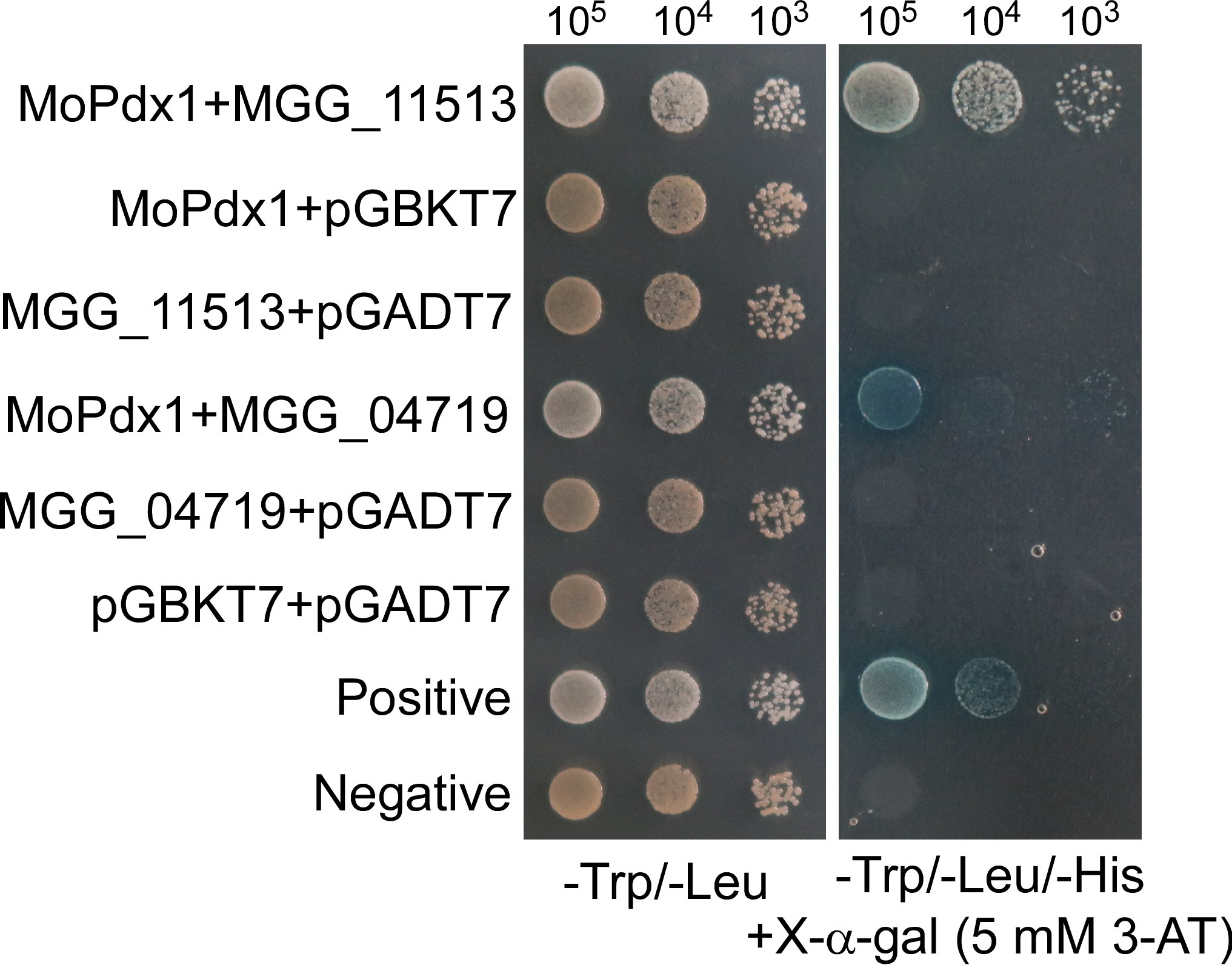

Supplement: Supplementary Figure 3 — Interaction of MoPdx1 with proteins encoded by MGG_11513 and MGG_04719 in a yeast two hybrid assay, respectively. MoPDX1 was inserted into vector pGADT7, and MGG_11513 or MGG_04719 were individually inserted into vector pGBKT7. The latter were co-transferred separately with the MoPDX1 vector into yeast AH109 cells and incubated on SD-Leu-Trp for 3 days, then selected on SD-Leu-Trp-His with 1 mM X-α-gal and 5 mM 3-AT (3-amino-1,2,4-triazole) for another 3 days. [file Image_3.tif]

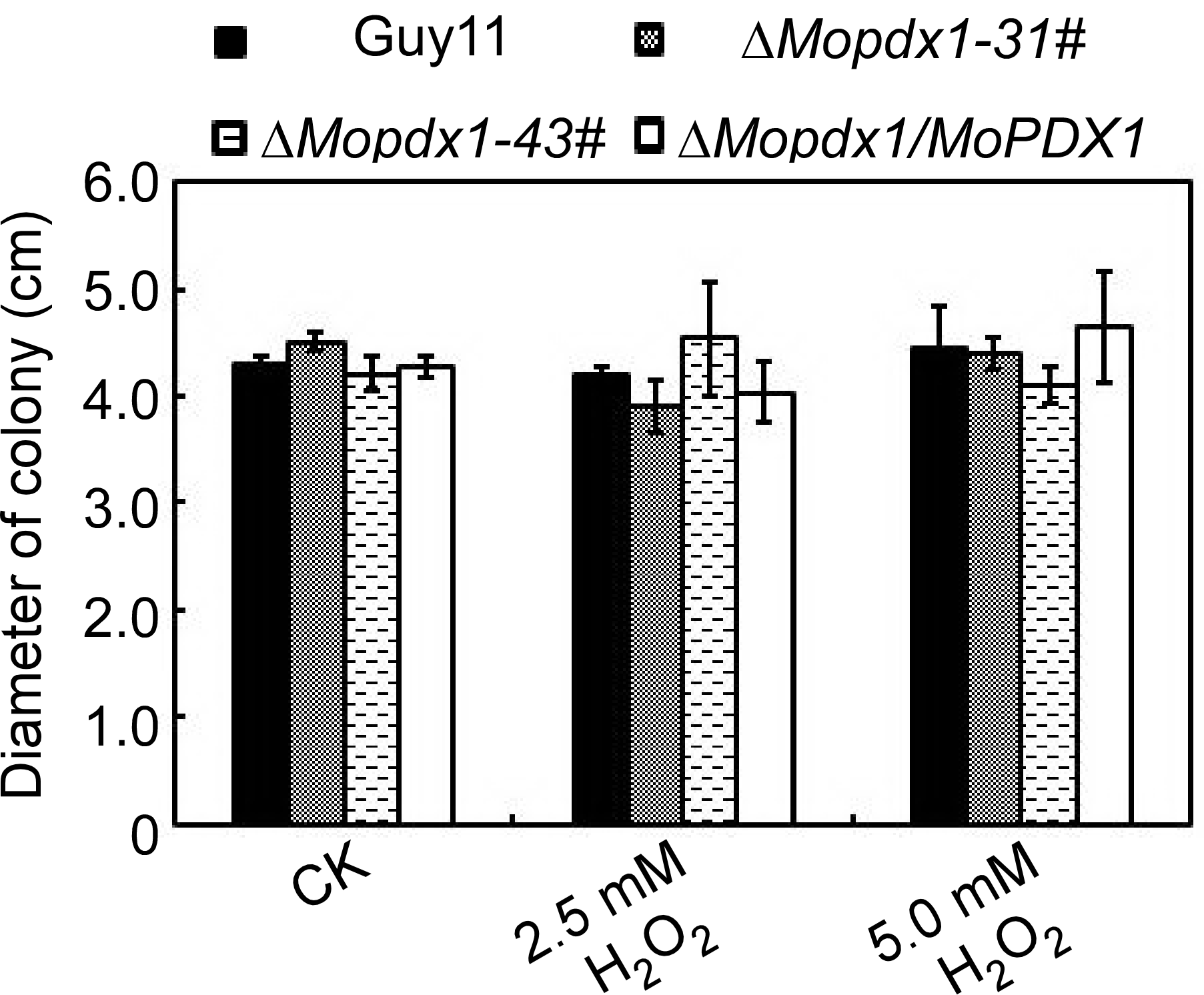

Supplement: Supplementary Figure 4 — MoPdx1 is not involved in the oxidative stress response of M. oryzae. Hyphal blocks of the indicated strains were cultured on CM media with or without 2.5 or 5.0 mM H2O2 at 28°C in the dark. The colony diameters were measured at 7 dpi. Error bars represent the standard deviation. There was no significant difference under the different treatments in indicated strains (LSD and Student-Newman-Keuls test, p<0.05). All experiments were performed for three independent replicates with the same results. [file Image_4.tif]
