# Supplementary material for: Pyridoxine biosynthesis protein MoPdx1 affects the development and pathogenicity of Magnaporthe oryzae
Source: Front Cell Infect Microbiol. 2023 Feb 7;13:1099967. doi: 10.3389/fcimb.2023.1099967 (PMC9941553; doi:10.3389/fcimb.2023.1099967)
Supplement: Supplementary Table 1 — Primers used in this study. [file Table_1.docx]

Table S1 Primers used in this study.

| **Primer name** | **Sequence (5’-3’)** | **Remark** |
| --- | --- | --- |
| *PDX1* KO-F1 | TAACTCGAG TACCGATACAAGATGCTGCACTC | amplify *MoPDX1* 5’ flank sequence |
| *PDX1* KO-F2 | TAAAAGCTTCTTTGATATTCGAATGAGGTAT | amplify *MoPDX1* 3’ flank sequence |
| *PDX1* KO-F3 | TAAGGATCCTGGGTCAACCCAGGTCTAGGC | amplify *MoPDX1* 5’ flank sequence |
| *PDX1* KO-F4 | TAAGAGCT'CTTTGGCTTCACTCAAGCCGAC | amplify *MoPDX1* 3’ flank sequence |
| *PDX1* BN-C1 | GACTTCGCAGTCCCCTTCGTG | Verification of *MoPDX1* deletion |
| *PDX1* BN-C2 | AACCGCCAGCTTCTCCGACTG | Verification of *MoPDX1* deletion |
| *PDX1* BY | GTGGCTCGTGGCCAATGCAGATA | Verification of *MoPDX1* deletion |
| *PDX1* HB-F | ACT CAC TAT AGG GCG AAT TGG GTA CTC AAA TTG GTT GTGGCTCGTGGCCAATGCAGATA | *MoPDX1* complementation |
| *PDX1* HB -R | CAC CAC CCC GGT GAA CAG CTC CTC GCC CTT GCT CAC CCATCCCCGAACCGCCAGCTT | *MoPDX1* complementation |
| *PDX1* HB -C | GACTTCGCAGTCCCCTTCGTG | Verification of *MoPDX1* complementation |
| HPH-probe-F | TCGTTATGTTTATCGGCACTTTG | amplify *HPH* probe 5’ flank sequence |
| HPH-probe-R | TGTTGGCGACCTCGTATTGG | amplify *HPH* probe 3’ flank sequence |
| RT-*PDX1*-F | TCGAGTGCGACCCTGCCCTGTT | qRT-PCR Primer of *MoPDX1* |
| RT-*PDX1*-R | ATACTCTGCAAGCATCTTTGCAT | qRT-PCR Primer of *Mo PDX1* |
| AD-*PDX1*-F | TAACATATGATGGCCTCGACAACTTCCAATG | Construction of pGADT7-*MoPDX1* |
| AD-*PDX1*-R | TAAGAATTCTTACCATCCCCGAACCGCCAGC | Construction of pGADT7-*MoPDX1* |
| Rubq1 LL | GTGGTGGCCAGTAAGTCCTC | quantitative RT-PCR analysis |
| Rubq1 RR | GGACACAATGATTAGGGATCA | quantitative RT-PCR analysis |
| 28S rDNA LL | TACGAGAGGAACCGCTCATTCAGATAATTA | quantitative RT-PCR analysis |
| 28S rDNA RR | TCAGCAGATCGTAACGATAAAGCTACTC | quantitative RT-PCR analysis |
| Rice_PBZ1_QF | CTACTATGGCATGCTCAAGAT | quantitative RT-PCR analysis |
| Rice_PBZ1_QR | ATAGAAAGGCACATAAACACAA | quantitative RT-PCR analysis |
| Rice_Cht1-F | CGTGGTGACCAACATCATCA | quantitative RT-PCR analysis |
| Rice_Cht1-R | GAGTTGAAAGGCCTCTGGTTGT | quantitative RT-PCR analysis |
| Rice_ EF1α_QF | CTTCAACACCCCTGCTATG | quantitative RT-PCR analysis |
| Rice_EF1α_QR | CCGTTGTGGTGAATGAGTAA | quantitative RT-PCR analysis |
