# Supplementary material for: Pyridoxine biosynthesis protein MoPdx1 affects the development and pathogenicity of Magnaporthe oryzae
Source: Front Cell Infect Microbiol. 2023 Feb 7;13:1099967. doi: 10.3389/fcimb.2023.1099967 (PMC9941553; doi:10.3389/fcimb.2023.1099967)
Supplement: Supplementary Table 2 — Measurement of the content of VB6 in different strains on CM media by HPLC-MS. [file Table_2.docx]

Table S2 Measurement of the content of VB6 in different strains on CM media by HPLC-MS.

| Strains | Peak time (min) | VB6 content  (pmol/mg mycelium) |
| --- | --- | --- |
| Guy11 | 1.258 | 155.7 ± 41.6a |
| Δ*Mopdx1-31#* | 1.275 | 163.3 ± 30.3a |
| Δ*Mopdx1-43#* | 1.272 | 139.6 ± 35.2a |
| Δ*Mopdx1/MoPDX1* | 1.271 | 185.3 ± 42.3a |

±SD was calculated from three repeated experiments and lowercase indicates statistically significant differences (LSD and Student-Newman-Keuls test, *p*<0.05).
